# Small Intestinal Complications of Intestinal Transplant-Associated Microangiopathy Post-stem Cell Transplantation

**DOI:** 10.7759/cureus.64111

**Published:** 2024-07-08

**Authors:** Daisuke Kametaka, Masaya Iwamuro, Takehiro Tanaka, Ken-ichi Matsuoka, Motoyuki Otsuka

**Affiliations:** 1 Department of Gastroenterology and Hepatology, Okayama University Graduate School of Medicine, Dentistry, and Pharmaceutical Sciences, Okayama, JPN; 2 Department of Pathology, Okayama University Hospital, Okayama, JPN; 3 Department of Hematology and Oncology, Okayama University Graduate School of Medicine, Dentistry, and Pharmaceutical Sciences, Okayama, JPN

**Keywords:** video capsule enteroscopy, stem cell transplantation, intestinal transplant-associated microangiopathy, graft-versus-host disease, cytomegalovirus infection

## Abstract

We present the case of a patient who underwent human leukocyte antigen-haploidentical transplantation for T-cell acute lymphoblastic leukemia. Seven weeks after transplantation, the patient developed intestinal transplant-associated microangiopathy (iTAM). Although the iTAM was resolved temporarily, it recurred. Video capsule enteroscopy revealed multiple erosions and shallow ulcers in the jejunum and ileum. To the best of our knowledge, this is the first report to present images of possible small intestinal lesions in iTAM. The small intestinal mucosal images presented herein may potentially aid in the management of similar patients.

## Introduction

Intestinal transplant-associated microangiopathy (iTAM) is a condition characterized by damage to the small blood vessels (microangiopathy) in the intestine following a transplantation procedure, particularly after stem cell transplantation for blood disorders [1-4]. This damage can lead to complications such as tissue injury, impaired blood flow, and, ultimately, organ dysfunction. iTAM is a severe complication that can significantly affect the success of the transplant and the overall health of patients [5]. Treatment for iTAM typically involves a multifaceted approach to address the underlying causes and manage symptoms. It is important to note that the treatment differs from other intestinal complications, such as graft-versus-host disease (GVHD) or cytomegalovirus infection, that occur after hematopoietic stem cell transplantation [6-8]. Therefore, appropriate diagnostic and treatment interventions for iTAM are crucial for managing post-hematopoietic stem cell transplantation recipients. Although iTAM can cause mucosal damage in the small intestine, endoscopic images of the small intestinal lesions have not yet been reported. Herein, we present the case of a patient in whom small intestinal lesions were visualized using video capsule enteroscopy.

## Case presentation

**Figure 1:**
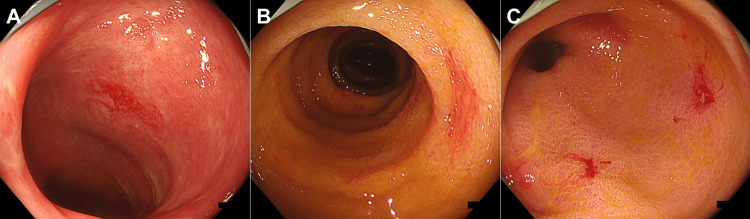
Colonoscopy images performed seven weeks after HLA-haploidentical transplantation (A) The mucosa of the ileum exhibits villous atrophy. (B and C) Edematous and coarse mucosa was observed throughout the entire colon, with erosions in the descending colon and multiple shallow ulcers in the rectum. HLA, human leukocyte antigen

**Figure 2:**
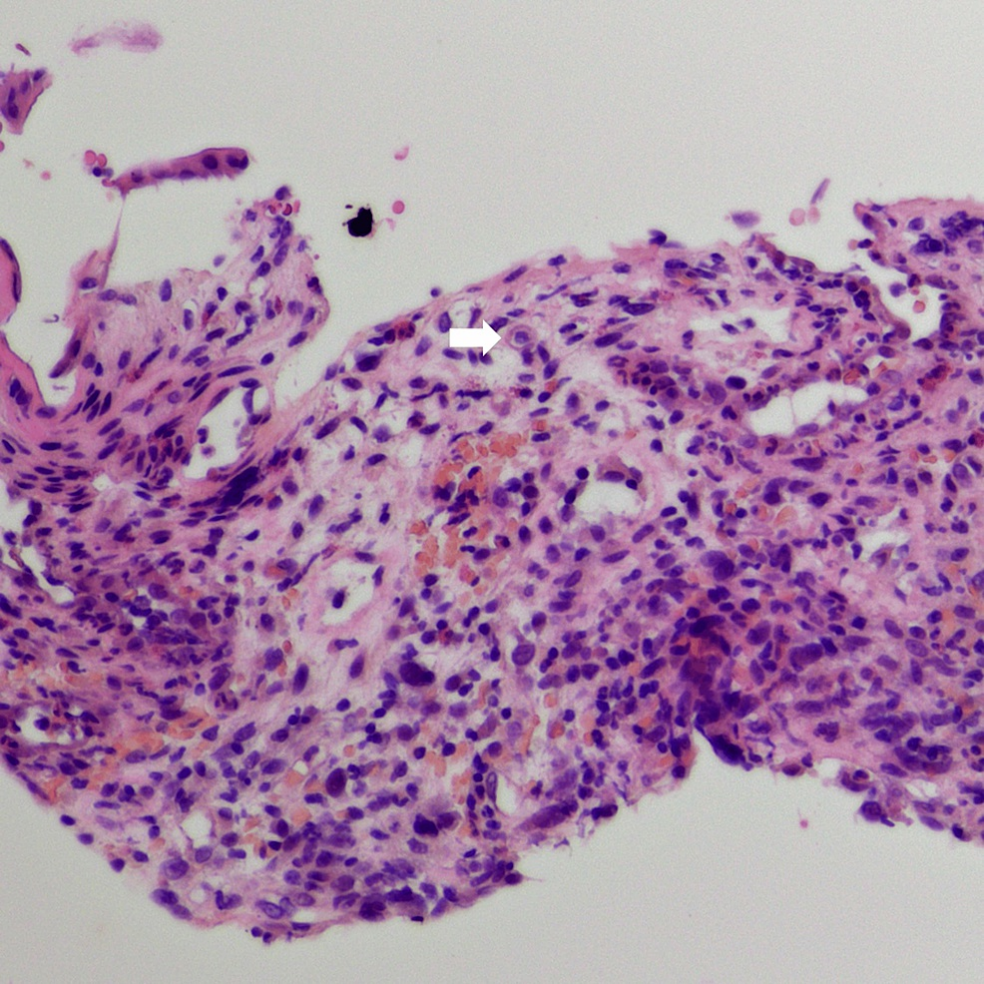
A pathology image of the colonic mucosa Hypertrophy of the vascular endothelium is observed, leading to a diagnosis of iTAM (arrow). iTAM, intestinal transplant-associated microangiopathy

A Japanese man was diagnosed with acute T-cell lymphoblastic leukemia at the age of 23. He experienced refractory relapse and underwent the following treatments: umbilical cord blood transplantation at the age of 25, human leukocyte antigen (HLA)-haploidentical transplantation with post-transplant cyclophosphamide at the age of 27, a second umbilical cord blood transplantation at the age of 28, and HLA-haploidentical transplantation with post-transplant cyclophosphamide at the age of 29. The patient was hospitalized seven weeks after the last transplantation because of increased diarrhea. A colonoscopy revealed villous atrophy of the ileal mucosa. Edematous and coarse mucosa were observed throughout the colon, with erosion in the descending colon and multiple shallow ulcers in the rectum (Figure 1). Histopathological examination of endoscopic biopsy samples from the ileum to the rectum revealed denudation and atrophy of epithelial cells with areas of fibrosis. Hypertrophy of the vascular endothelium was also observed, leading to a diagnosis of iTAM (Figure 2). Additionally, a small number of cells tested positive for cytomegalovirus on immunohistochemical staining, suggesting concurrent cytomegalovirus enteritis. Tacrolimus was discontinued based on the diagnosis of iTAM. Ganciclovir was administered intravenously for cytomegalovirus enteritis.

**Figure 3:**
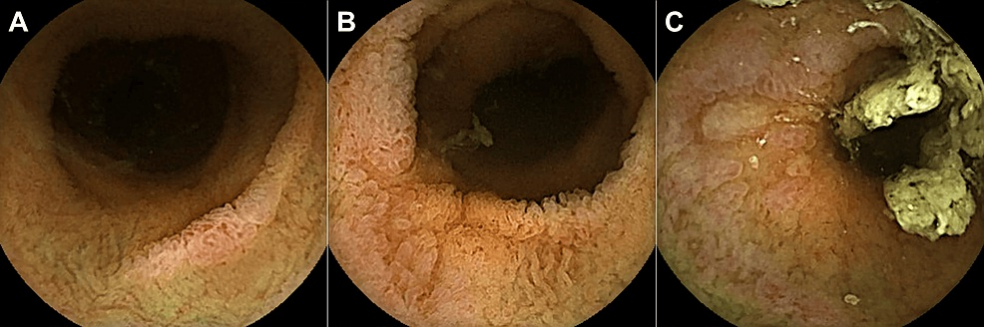
Video capsule enteroscopy images Multiple erosions and shallow ulcers are observed mainly on Kerckring’s folds of the jejunum and ileum.

Additionally, prednisolone (1 mg/kg body weight) was administered to treat suspected concurrent GVHD. At 24 weeks post-transplantation, colonoscopy showed an improvement in mucosal damage, with no histopathological findings of iTAM or the presence of cytomegalovirus-positive cells. The patient was discharged; however, 30 weeks post-transplantation, he presented with watery diarrhea and bloody stools, leading to readmission. A colonoscopy revealed scattered erythematous erosions throughout the colon, which were suspected to be the source of the bleeding. A biopsy revealed segmental damage with epithelial apoptosis, loss of glands, and total mucosa denudation, indicating iTAM recurrence. Video capsule enteroscopy revealed multiple erosions and shallow ulcers, mainly in the folds of the jejunum and ileum (Figure 3). The blood test results at the time of the video capsule enteroscopy are shown in Table 1. Subsequently, the patient participated in a clinical trial of antibody therapy.

**Table 1:** Blood test results

Blood test results (units)	Patient value	Reference range
White blood cells (/µL)	3,440	3,300–8,600
Neutrophils (%)	32.5	40–70
Lymphocytes (%)	35	16.5–49.5
Monocytes (%)	28.5	2–10
Hemoglobin (g/dL)	7.9	11.6–14.8
Schistocytes (%)	2.8	
Platelets (/µL)	1.6 × 10^4^	15.8 × 10^4^–34.8 × 10^4^
Total protein (g/dL)	4.9	6.6–8.1
Albumin (g/dL)	2.5	4.1–5.1
Creatinine (mg/dL)	0.74	0.46–0.79
Lactate dehydrogenase (U/L)	167	124–222
Sodium (mmol/L)	136	138–145
Potassium (mmol/L)	3.9	3.6–4.8
Aspartate aminotransferase (U/L)	19	13–30
Alanine aminotransferase (U/L)	32	7–2
γ-Glutamyl transpeptidase (U/L)	176	9–32
C-reactive protein (mg/dL)	1.77	0–0.15
D-dimer (µg/mL)	12.3	0–0.9
Haptoglobin (mg/dL)	203	19–170

## Discussion

Endoscopic images of iTAM have not been sufficiently reported. Yamada-Fujiwara et al. reported nine cases of iTAM, of which six were concurrent with GVHD. They described mucosal petechiae and diffuse exfoliation as the criteria for the clinical diagnosis of iTAM based on colonoscopic findings [9]. Yamamoto et al. presented an endoscopic image of a case showing segmental longitudinal ulcerations with hemorrhagic edematous mucosa in the transverse colon, suggesting that iTAM may present endoscopic images resembling ischemic colitis [10]. In a previous study by Yamada et al., four patients were diagnosed with iTAM [11]. Concomitant GVHD was consistently observed in all the cases. Endoscopic examination revealed erythema and edema, along with erosions and ulcerations. Another investigation by Nishida et al. involving nine patients with concurrent iTAM and GVHD documented a range of gastrointestinal tract anomalies, notably mucosal edema, erythema, ulceration, and hemorrhage [12]. In light of the limited reports on endoscopic imaging of iTAM, we recently conducted a comprehensive investigation and reported the endoscopic findings in 14 cases of iTAM [13]. All the patients had erythematous mucosa in the gastrointestinal tract. Endoscopic findings in iTAM included erosions (93%), ulcers (64%), mucosal edema (64%), granular mucosa (64%), and villous atrophy (29%).

To date, endoscopic images of small intestinal lesions have not been reported, although iTAM can manifest as lesions in the small intestine [11]. This is the first report to present video capsule enteroscopy images of possible jejunal and ileal iTAM lesions. GVHD is characterized by extensive lymphocyte infiltration within the mucosa and infiltration of CD8+ T lymphocytes into the epithelium, along with apoptosis within the crypts [14], resulting in diffuse rather than regional changes in the gastrointestinal mucosa. In contrast, iTAM involves damage to small blood vessels (microangiopathy) in the intestine, leading to ischemia and subsequent wedge-shaped segmental tissue injury [3,4,15]. Therefore, the multiple erosions and shallow ulcers observed in this case, without extensive mucosal injuries such as edema, erythema, and villous atrophy, are consistent with small intestinal lesions of iTAM.

There are some limitations to our study that may impact the interpretation of the small intestinal lesions. Firstly, there are no established pathologic criteria for diagnosing iTAM. Secondly, histological diagnosis via biopsy was not performed for small intestinal lesions in this case due to obtaining images through video capsule enteroscopy. It is noteworthy that GVHD, cytomegalovirus infection, and iTAM can co-occur. Specifically, cytomegalovirus-positive cells were detected in the colorectum during the course of this case, and steroid treatment was initiated, suspecting GVHD. Consistent with our previous report, we diagnosed iTAM of the colorectum based on hypertrophy of the vascular endothelium (Figure 2). However, due to the patient not meeting the symptoms of TA-TMA [16] and the lack of established pathologic criteria, we cannot definitively assert whether the small intestinal lesions are iTAM or related to GVHD or cytomegalovirus infection in the small intestine. However, post-HSCT patients often have compromised overall health, and performing balloon-assisted enteroscopy to obtain small intestinal tissue for diagnosis carries a significant risk. Therefore, small intestinal mucosa evaluation using video capsule enteroscopy is a less invasive option for gastrointestinal assessment in post-hematopoietic stem cell transplant patients. Thirdly, the clinical significance of small intestinal lesions has not been clearly established. Recently, within TA-TMA, attention has focused on high-risk, complement-mediated, untreated transplant-associated thrombotic microangiopathy, which leads to dismal outcomes due to multi-organ dysfunction syndrome [17]. This case not only fails to meet the criteria for high-risk TA-TMA but also does not meet the standards for TA-TMA [16], indicating a clinically mild severity. The impact of small intestinal lesions on the clinical course of iTAM and the types of small intestinal lesions observed in severe cases remain unclear. Addressing these issues will require a further accumulation of cases. Nonetheless, this report represents the first presentation of endoscopic images of possible small intestinal lesions in iTAM, and we anticipate that the mucosal images presented here will contribute to the management of similar patients.

## Conclusions

We present endoscopic images of possible small intestinal lesions in iTAM, demonstrating multiple erosions and shallow ulcers in the jejunum and ileum. To the best of our knowledge, this report is the first to present endoscopic images of small intestinal lesions associated with iTAM. Intestinal diseases that occur in patients post-hematopoietic stem cell transplantation include iTAM, GVHD, and cytomegalovirus infection. Given the distinct treatment approaches required for these conditions, precise diagnostic and therapeutic interventions are crucial for the effective management of patients following hematopoietic stem cell transplantation. Video capsule enteroscopy may help detect small intestinal involvement in post-hematopoietic stem cell transplant recipients with iTAM.
